# Type II Collagen Sponges Facilitate Tendon Stem/Progenitor Cells to Adopt More Chondrogenic Phenotypes and Promote the Regeneration of Fibrocartilage-Like Tissues in a Rabbit Partial Patellectomy Model

**DOI:** 10.3389/fcell.2021.682719

**Published:** 2021-07-16

**Authors:** Wen Wang, Shengnan Qin, Peiliang He, Wei Mao, Liang Chen, Xing Hua, Jinli Zhang, Xifeng Xiong, Zhihe Liu, Pengzhen Wang, Qingqi Meng, Fei Dong, Aiguo Li, Honghui Chen, Jiake Xu

**Affiliations:** ^1^Department of Orthopedics, Guangzhou Institute of Traumatic Surgery, Guangzhou Red Cross Hospital, Medical College, Jinan University, Guangzhou, China; ^2^Department of Clinical Medicine, Guizhou Medical University, Guiyang, China; ^3^Department of Pathology, Guangzhou Red Cross Hospital, Medical College, Jinan University, Guangzhou, China; ^4^School of Biomedical Sciences, The University of Western Australia, Perth, WA, Australia

**Keywords:** tendon stem and progenitor cells, type II collagen sponges, fibrocartilage transition zones, bone tendon junction, chondrogenesis

## Abstract

**Objective:**

Fibrocartilage transition zone (FC) is difficult to regenerate after surgical re-attachment of tendon to bone. Here, we investigated whether type II collagen-sponges (CII-sponges) facilitated tendon stem/progenitor cells (TSPCs) to adopt chondrogenic phenotypes and further observed if this material could increase the FC areas in bone-tendon junction (BTJ) injury model.

**Methods:**

CII-sponges were made as we previously described. The appearance and pore structure of CII-sponges were photographed by camera and microscopies. The viability, proliferation, and differentiation of TSPCs were examined by LIVE/DEAD assay, alamarBlue, and PKH67 *in vitro* tracking. Subsequently, TSPCs were seeded in CII-sponges, Matrigel or monolayer, and induced under chondrogenic medium for 7 or 14 days before being harvested for qPCR or being transplanted into nude mice to examine the chondrogenesis of TSPCs. Lastly, partial patellectomy (PP) was applied to establish the BTJ injury model. CII-sponges were interposed between the patellar fragment and tendon, and histological examination was used to assess the FC regeneration at BTJ after surgery at 8 weeks.

**Results:**

CII-sponges were like sponges with interconnected pores. TSPCs could adhere, proliferate, and differentiate in this CII-sponge up to 14 days at least. Both qPCR and immunostaining data showed that compared with TSPCs cultured in monolayer or Matrigel, cells in CII-sponges group adopted more chondrogenic phenotypes with an overall increase of chondrocyte-related genes and proteins. Furthermore, in PP injured model, much more new formed cartilage-like tissues could be observed in CII-sponges group, evidenced by a large amount of positive proteoglycan expression and typical oval or round chondrocytes in this area.

**Conclusion:**

Our study showed that CII-sponges facilitated the TSPCs to differentiate toward chondrocytes and increased the area of FCs, which suggests that CII-sponges are meaningful for the reconstruction of FC at bone tendon junction. However, the link between the two phenomena requires further research and validation.

## Introduction

Fibrocartilage transition zone (FC) is the structure of bone tendon junction. Surgical reattachment of tendon to bone often fails due to the lack of regeneration of this specialised structure, thereby presenting difficulty for tendon to bone healing. It is reported that many factors, like mechanical loading, extracellular matrix, and biological factors (Wong et al., 2004; Lui P. et al., 2010; Hu et al., 2015; Leung et al., 2015), may contribute to better healing outcome at the junction between tendon graft and bone. However, it is important to note that the new-formation of cartilaginous tissues between the two completely different or inhomogeneous tissues is a key process even though these tissues function as intermediate in endochondral ossification and disappear with time during healing eventually. It is confirmed that the reconstruction from patellar tendon to cartilage was much easier to that from tendon to the remaining patellar in partial patellectomy model (Lu et al., 2008). Several studies have also demonstrated that cartilaginous tissues formed between tendon graft and bone, resembling the natural transition zone in ACL reconstruction surgery (Yamakado et al., 2002; Lim et al., 2004; Park et al., 2018). Many studies reported better healing outcomes with the appearance of chondrocyte-like cells between tendon graft and tendon (Grassman et al., 2002; Wang et al., 2010, 2014; Hu et al., 2015; Lu et al., 2015, 2016; Song et al., 2017). Wong et al. (2009) showed that articular cartilage interposed in patellar tendon and the remaining patellar could result in more FC than direct repair at all time points, and hence stronger mechanical strength recovery. These findings suggest that the formation of cartilage interface by different means could be an effective way to promote the regeneration of native FC and hence be beneficial to the reconstruction of bone tendon junction.

Considering that the implication of cartilaginous interfaces for regeneration of bone tendon junction, it is important to induce stem/progenitor cells from either bone marrow or tendon to differentiate toward chondrocytes or fibrochondrocytes after tendon reconnected to bone tissues. Our previous study showed that tendon stem/progenitor cells (TSPCs) were differentiated toward fibrochondrocytes under classical chondrogenic induction medium, so TSPCs could be candidate for the seed cells for bone tendon junction repair (Qin et al., 2020). However, in our previous study, only a proportion of TSPCs after induction differentiated, and the reason for this might be due to the fact that chondrocytes in monolayer 2D culture dedifferentiate to a less specialised fibroblastic phenotype and produce less cartilage extracellular matrix (ECM). The ECM on which stem cells grow has been proved to play a particularly important role in controlling cell differentiation fate (Badylak, 2005; Evans et al., 2010). Type II collagen is the main component of articular cartilage of knee, and scaffolds made of type II collagen have been proved to direct stem cells toward chondrocytes and promote the repair of cartilage defect (Chen et al., 2011). Compared to monolayer, chondrocytes grown in 3D porous collagen sponges can maintain the expression of chondrocyte-specific genes as well as the production of cartilage-specific ECM (Yates et al., 2005).

The aim of this study is to induce TSPCs to adopt more chondrogenic phenotype, we chose cross-linked type II collagen-sponges (CII-sponges) which we previously homemade to provide a three-dimensional environment for TSPCs and further observed the role of this material in the regeneration of FCs at bone-tendon junction (BTJ). The CII-sponges have been proved to be beneficial to the new formation of cartilage-like tissues at the site of full-thickness articular cartilage defect as evidenced by positive proteoglycan staining and positive type II collagen staining (Chen et al., 2011). Here, we observed the chondrogenic differentiation of TSPCs in the pellet culture system. We also assessed the growth of cells that were seeded in our homemade CII-sponges and further investigated the role of CII-sponges in directing TSPCs toward chondrocytes or fibrochondrocytes. Finally, the regeneration of FCs at injured bone tendon junctions was evaluated. We show that CII-sponges effectively directed TSPCs differentiation toward fibrochondrocytes, and also could increase the formation of fibrocartilage-like tissues at the BTJ.

## Materials and Methods

### Cell Culture

The TSPCs were isolated from human tendon as previously described (Qin et al., 2020). Cells at passage 6 were employed to do experiments.

### Immunocytochemistry Staining

To observe the chondrogenic proteins expression of human TSPCs, cells were harvested and seeded in coverslips. Cells were fixed using 4% paraformaldehyde for 10 min at room temperature before the immunocytochemistry (ICC) procedure. The samples were incubated with PBS containing 0.25% Triton X-100 to permeabilise the cells. In order to block unspecific binding of the antibodies, cells were incubated with 1% BSA in PBS for 30 min, and then incubate cells with the diluted primary antibodies, including Collagen I, Collagen II, Sox9, and Aggrecan, in 1% BSA in PBS for overnight at 4°C. Subsequently were incubated the cells in 0.3% H_2_O_2_ to block the endogenous peroxidase, and then applied to the secondary antibodies for 1 h at room temperature. Finally, the samples were developed with chromogen for 3 min at room temperature, rinsed with in running tap water for 5 min and counterstained with haematoxylin.

### The Chondrogenic Induction of Tendon Stem Cells in Pellet Culture System

To assess the chondrogenic differentiation potential of TSPCs we isolated, classical pellet culture system was used. TSPCs (passage 6) were centrifuged and then cultured in chondrogenic induction medium which is LG-DMEM with 10% FBS and containing 10^–7^ M dexamethasone, 40 μg/ml proline, 100 μg/ml pyruvate, 50 μg ascorbate-2-phosphate (all from Sigma-Aldrich), and 1:100 diluted ITS + premix (6.25 mg/ml insulin, 6.25 mg/ml transferrin, 1.25 mg/ml bovine serum albumin, 5.35 mg/ml linoleic acid, and 6.25 mg/ml selenous acid) (Becton Dickinson, Franklin Lakes, NJ, United States) as well as TGF-β3/BMP-2 (10 ng/ml TGF-β3 and 50 ng/ml BMP-2). After 21 days, pellets were harvested to be fixed and embedded in order to observe the expression of proteins mentioned above using by immunohistochemistry staining.

### The Preparation of CII-Sponges

Type II collagen was extracted as previously described (Chen et al., 2011). Type II collagen protein was extracted from the hyaline cartilage of knees from pigs. Firstly, the hyaline cartilage was peeled off and cut into pieces. Secondly, type II collagen fibres were dissolved with acetic acid solution containing pepsin into solution and centrifuged in order to remove those undissolved cartilage tissues. Thirdly, the supernatant with type II collagen was mixed with NaCl solution in a way collagens dissolved into acetic acid can be salted out. Subsequently, these salted-out collagens were re-dissolved in acetic acid to get the purified collagen solution. Finally, this purified collagen solution were re-adjusted by dialysing with distilled water to PH 5.5.

In order to get CII-sponges, two steps were applied. First, the purified collagen solution per well was poured into 24-well plate in every 0.5 ml solution per well, and after freeze-drying by vacuum freeze-dryer (PINPAI, United States), sponge-like materials were obtained. Secondly, due to the fact that collagen sponges we got in the last step would be dissolved in water easily, the collagen sponges needed to be cross-linked before being used as scaffolds. In order to get cross-linked CII-sponges, these CII-sponges were further cross-linked with EDC/NHS as we previously described (Qin et al., 2010).

### Pore Structure of CII-Sponges

The appearance of the CII-sponges was photographed by camera, and the pore structure and porosity of the CII-sponges were observed by laser confocal electron microscopy (LCM), scanning electron microscopy (SEM), and bright field microscopy (BFM). For LCM, collagen has autofluorescence, thereby producing a bright green fluorescence in sponges with the excitation wavelength of 488 nm and the emission wavelength of 520 nm using the Confocal microscope, so the pore diameter in the same layer could be seen. For SEM examination, the samples were air dried, sputter-coated with gold and the pore structure could be observed. The samples were also observed by microscopy under BFM.

### TSPCs Seeded in Monolayer, Matrigel, or CII-Sponges

TSPCs were seeded in monolayer, Matrigel, or CII-sponges for 7 or 14 days with or without chondrogenic induction. For monolayer, 10^6^ TSPCs were seeded in 10 cm culture dish. For Matrigel, 10^6^ TSPCs were collected and resuspended in 0.2 ml medium, and then mixed with 0.2 ml Matrigel. After several seconds, Matrigel could form gel-like scaffold. For CII-sponges, 10^6^ cells were resuspended in 0.5 ml medium and then seeded in 2.5 cm^2^ CII sponge. Matrigel- or CII sponge-cells compounds were then cultured in 24 well plates.

### The Viability of TSPCs Seeded in CII-Sponges

LIVE/DEAD assay was used to check the cell viability of TSPCs after 14 days culture. TSPCs at passage 6 were harvested and approximately 10^6^ cells were seeded in each 2.5 cm^2^ CII-sponges. After 3-day culture, the sponges were changed to a new dish in order to rule out the cells which did not adhere to the materials. Meanwhile, the culture medium was changed to chondrogenic medium. Cells viability was checked by LIVE/DEAD assay (ThermoFisher, United States) in the process of chondrogenic induction, and live/dead cells after 14-day induction were photographed by immunofluorescence microscope. The sponges with cells were washed once by PBS in order to remove the serum in culture medium, and then 1 ml PBS with 2 μl Calcein AM and 0.4 μl EthD-1 was added in each sponges. Meanwhile, one drop of NucBlue^TM^ Live ReadyProbes^TM^ Reagents (ThermoFisher, United States) was added per 1 ml PBS to distinguish the nuclei in cells. After 30 min incubation at room temperature, live cells could retain the polyanionic dye calcein AM well, thereby producing a bright green fluorescence at the excitation/emission wavelength ∼495/∼515 nm. At the same time, EthD-1 could enter dead cells and produce an intense strong red fluorescence by binding to nucleic acids at the excitation/emission wavelength ∼495/∼635 nm. NucBlue^TM^ Live cell stain was excited by UV light, emitting blue fluorescence at the excitation/emission wavelength ∼360/∼460 nm.

AlamarBlue Cell Viability Regent (ThermoFisher, United States) was used to monitor the proliferation of TSPCs seeded in CII-sponges without compromising cell health. The viability of TSPCs seeded in CII-sponges was measured at day 1, 3, 7, and 14, respectively. To assay for viability, the pre-mixed alamarBlue reagent were added with the completed medium to cells, incubated for 2 h, and absorbance values were measured using a plate reader (BioTek, United States).

### *In vitro* Tracking of TSPCs Seeded in CII-Sponges

PKH67 kit is for general cell membrane labelling, and can be applied to label and track cells over an extended period of time *in vitro*, with no apparent toxic effects. Due to the fact that non-dividing cells remain brightly labelled with membrane intercalating dyes and the same stem cell could not proliferate and differentiate simultaneously, we examined the labelled cells remaining in CII-sponges with or without chondrogenic induction *in vitro*. TSPCs were labelled with PKH67 according to manufacturers’ protocol, and seeded in CII-sponges. The collagen sponges seeded with cells were cultured in completed medium for 7 days, and then would continue to be cultured in completed medium or induced with chondrogenic medium for further 14 days. TSPCs labelled with PKH67 would be observed with fluorescence microscopes at day 7 and 21.

### q-PCR

Cells were seeded in monolayer, Matrigel, and CII-sponges and continued to be cultured in completed medium for 7 days. Subsequently, cells were cultured in chondrogenic medium or completed medium for 7 or 14 days. Total RNA was extracted using TRIzol^TM^, and cDNA was synthesised using 500 ng RNA with PrimeScript RT master Mix (Takara, China) according to manufacturer’s instruction. The resulting cDNA was then used for real-time PCR with PowerUP^TM^ SYBR^TM^ Green Master Mix (ThermoFisher, United States) by Analytik Jena qTOWER^G^. The conditions for PCR were as follow: 50°C for 2 min, 95°C for 5 min, followed by 40 cycles of (95°C for 15 s, 60°C for 60°C 1 min) with a melt curve stage of (95°C for 15 s, 60°C for 1 min, 95°C for 15 s). The primers used were as follow: Col1α1 (Forward: 5′-CAGCCGCTTCACCTACAGC-3′, Reverse: 5′-TTTTGTATTCAATCACTGTCTTGCC-3′), Sox 9 (Forward: 5′-TACGACTGGACGCTGGTGCC-3′ and Reverse: 5′-CCGTTCTTCACCGACTTCCTCC-3′), Col2α1 (Forward: 5′-GGCAATAGCAGGTTCACGTACA-3′ and Reverse: 5′-CGATAACAGTCTTGCCCCACTT-3′), Aggrecan (Forward: 5′-AAGTATCATCAGTCCCAGAATCTAGCA-3′ and Reverse: 5′-TTGGTGGAGACGTAAGGTGC-3′), and GAPDH (Forward: 5′-CGTAAAGACCTCTATGCCAACA-3′ and Reverse: 5′-CGGACTCATCGTACTCCTGCT-3′). The relative mRNA levels of all genes were normalised to housekeeping gene GAPDH and calculated using 2^–△^^△^^CT^ method.

### Heterotopic Chondrogenesis of TSPCs *in vivo*

The chondrogenic characteristics of TSPCs seeded in monolayer, Matrigel, or CII-sponges were observed *in vivo*. For monolayer, 10^6^ TSPCs were cultured in completed medium for 7 days in 10 cm dish. Meanwhile, 10^6^ TSPCs were seeded in Matrigel or collagen II sponge and also cultured in completed medium for 7 days. Cells in monolayer culture were then centrifuged to form a pellet due to the fact that cells without scaffolds were not able to be transplanted subcutaneously into the dorsal surface of immunocompromised nude mice. Cells in all of three groups were then induced by chondrogenic medium for 14 days *in vitro* before being transplanted. These transplants were harvested after 4 weeks, and proteoglycan or some proteins were further observed by histology examination.

### Partial Patellectomy Model

Twelve mature female New Zealand White rabbits (16 weeks old) underwent partial patellectomy and surgical reconstruction between the patella and patellar tendon using a previously established protocol (Wang et al., 2007). Briefly, with the animal under general anaesthesia with sodium pentobarbital, and the distal one-third of the patella as well as its fibrocartilage zone to the patellar tendon were removed. The patellar tendon was then directly sutured to the remaining proximal patella via the two holes drilled longitudinally through patella, with non-absorbable suture. CII-sponges were then interposed between patellar and patellar tendon. Direct repair was set as a control group. The operated knee joint was then immobilised for up to 4 weeks. Animals were kept individually in metal cages and fed with standard rabbit diet and water *ad libitum*. After 8 weeks, the animals were euthanised with an overdose of sodium pentobarbital, and the patellar and patellar tendon (PPT) complexes of the operated were harvested and prepared for histology examination. This study was approved by the Animal Research Ethics Committee of the Guangzhou Red Cross Hospital Ethics Committee (Approval No. 2018-002-01).

### Histology Examination

All samples, including pellet formed *in vitro*, and transplants harvested from nude mice and PPT complexes from rabbits, were immediately fixed in 10% neural formalin for 3 days, and subsequently PPT complexes were decalcified by 10% formic acid for more than 1 month. Samples were finally embedded in paraffin and sliced up at 5 μm. The sections were stained with Safranin O/Fast Green.

Immunohistochemical (IHC) staining were used to detect the expression of proteins, including Collagen I and II, Sox9, and Aggrecan (All from Abcam, United States), in continuous sections. The IHC staining was performed using a Mouse and Rabbit Specific HRP/DAB detection IHC kit (from ZSGB-BIO, China) according to manufacturer’s protocol. Briefly, deparaffinize and rehydrate the paraffin-embedded sections. Wash the slides in TBS plus 0.025% Triton-X100. After blocking with blocking solution of the kit, apple primary antibodies diluted in TBS with 1% BSA and allow the sections to incubate overnight at 4°C. Wash twice, and then block non-specific background staining. Apple biotinylated goat anti-rabbit/mouse antibody supplied with the kit and incubate for 15 min at room temperature. Apply streptavidin biotin peroxidase and incubate for 10 min at RT. Rinse four times, and add DAB substrate for less than 3 min to develop colour. Wash three times in buffer and apply H&E counterstain, dehydrate, and tissue clear. Meanwhile, in order to quantify the proteoglycan and proteins expression, the positive area of proteoglycan and all of proteins were analysed by ImageProPlus. To be more specific, the positive areas of proteoglycan (red) and proteins (brown) were counted by Image ProPlus, and the percentage of positive areas were normalised to the total areas of transplant from nude mice.

### Statistical Analysis

All data were presented as mean ± SD, and all reported representative data were from at least three independent experiments. All statistical analyses were performed with one-way AVONA analysis by PRISM 8. *p* ≦ 0.05 was considered as statistically significant. ns, represented as no significance; ^∗^ represented as *P* < 0.05, ^∗∗^ represented as *P* < 0.01, and ^∗∗∗^ represented as *P* < 0.001.

## Results

### Chondrocyte-Related Protein Expression of TSPCs and Chondrogenic Differentiation of TSPCs in Pellet Culture

**FIGURE 1:**
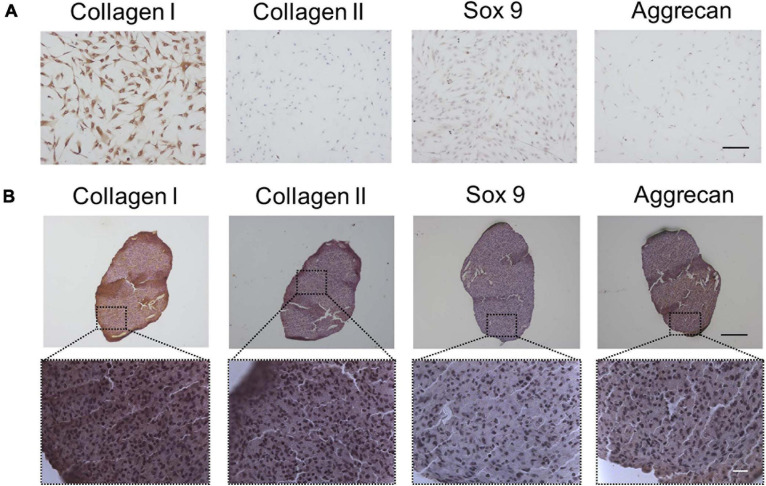
The fibrochondrocytes-related proteins expression of TSPCs by ICC or IHC staining. **(A)** TSPCs were cultured in completed medium in monolayer for 24 h, scale bar 200 μm. **(B)** TSPCs were induced in chondrogenic medium in pellet culture for 21 days, and continous slices were used to detect these proteins expression. Scale bar 100 μm (top) and 20 μm (bottom).

In order to determine whether TSPCs express chondrogenic differentiation proteins, Sox 9, collagen I, collagen II, and aggrecan expressions were investigated by ICC staining firstly. Strong expressions of collagen I in TSPCs were observed, while no expression of chondrocyte-specific proteins, including Sox 9, collagen II, and aggrecan, could be detected (Figure 1A).

Given we found that TSPCs differentiated toward fibrochondrocytes in monolayer culture, we chose the pellet culture system, a classic evaluation model for chondrogenesis of MSCs *in vitro*, to assess the chondrogenic differentiation potential of TSPCs from human tendon. IHC staining showed that after chondrogenic induction in pellet culture, Collagen I, a protein that was identified as the unique phenotype of TSPCs (Bi et al., 2007) and also as the fibroblast-specific protein, was still strongly expressed. However, aggrecan and type II collagen were weakly expressed, whereas Sox 9 was little expressed (Figure 1B).

### The Characterisation of CII-Sponges and Cell Viability as Well as *in vitro* Differentiation Tracking of TSPCs

**FIGURE 2:**
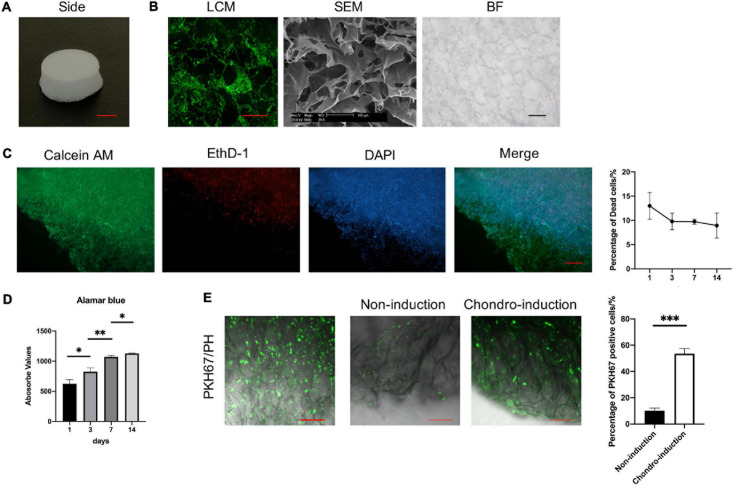
The characterisation of CII-sponges. **(A)** The appearance of CII-sponges, scale bar 0.5 cm. **(B)** The pore structure of CII-sponges scanned by laser confocal (LCM), scanning electricity (SEM), and bright field (BF) Microscopes, scale bar 200 μm (LCM), 100 μm (SEM), and 200 μm (BF). **(C)** The cell viability of TSPCs seeded in CII-sponges was detected by LIVE/DEAD assay, live cells (Green), dead cells (Red), and Nuclei (Blue), scale bar 200 μm. The percentage of dead cells in total cells at day 1, 3, 7, and 14 were analysed by Image ProPlus. **(D)** AlamarBlue detected the proliferation of TSPCs cultured in CII-sponges. **(E)** *In vitro* tracking of TSPCs labelled with PKH67 in CII-sponges. Scale 100 μm. The percentage of PKH67 positive cells with or without chondrogenic induction was qualified by Image ProPlus. * represented as *p* ≦ 0.05, ** represented as *p* ≦ 0.01, and *** represented as *p* ≦ 0.001.

CII-sponges we homemade looks like a porous sponge (Figure 2A). The pore structure of CII-sponges was examined by the LCM, the SEM, and BFM (Figure 2B). After layered scanning of LCM, aperture size in the same layer could be seen, and in our previous paper the size was 92.17 ± 29.55 μm (Qin et al., 2010), which was believed the appropriate pore size for chondrocytes growth (Nehrer et al., 1997; Zhang et al., 2014). SEM showed that CII-sponges had the film-like structures which are beneficial to cell adhesion and proliferation, and BF showed that this collagen sponge had similar aperture size and a large number of pores, which can be helpful to nutrient exchange. We also confirmed whether TSPCs could survive in this homemade collagen sponges after a period of culture *in vitro* (Figure 2C). The viability of TSPCs adhere to collagen scaffolds was assessed by LIVE/DEAD assay. It could be seen that the number of dead cells was decreased in a time manner, and more than 90% of the total cells were alive (Green), and a few of dead cells (red) could also be found after 14 days culture *in vitro*. AlamarBlue was used to track the proliferation of TSPCs adhere to CII-sponges, cells could proliferate well in the CII-sponges evidenced by the viability of cells enhanced over time (Figure 2D). In order to track the differentiation of TSPCs seeded in scaffolds, PKH67 was applied for labelling and tracking TSPCs (Figure 2E). Through multi-layer scanning using LCM, CII-sponges, and cells labelled with PKH67 could be observed at the same time through different channels. Cells labelled with PKH67 could produce green fluorescence, and CII-sponges were like as fibres by phase contrast (PH). After 7-day culture, cells adhere to materials could be found by green fluorescence. However, there was a difference in the number of cells with green fluorescence after a further 14-day culture with or without chondrogenic induction. A decrease in fluorescence was found in cells that continue to be cultured in completed medium, while a relatively slow decrease was found in cells under chondrogenic medium. More cells producing fluorescence could be seen in chondrogenic induction group than those in non-induction group.

### Chondrogenic Genes Expression of TSPCs in CII-Sponges

**FIGURE 3:**
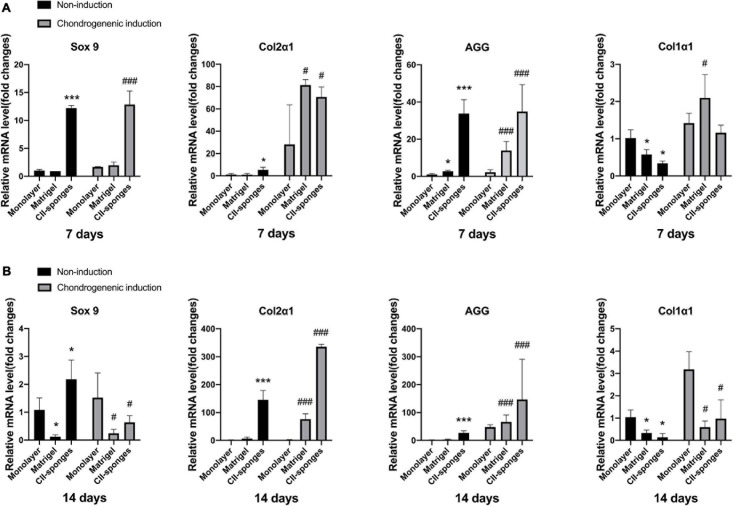
The fibrochondrocytes related genes expression with or without chondrogenic induction. **(A)** The mRNA levels of chondrogenic genes, including Sox 9, Col2α1 and AGG, and fibroblastic gene, Col1α1, in TSPCs seeded in monolayer, Matrigel, or CII-sponges for 7 days with or without chondrogenic induction. **(B)** The mRNA levels of chondrogenic genes, including Sox 9, Col2α1 and AGG, and fibroblastic gene, Col1α1, in TSPCs seeded in monolayer, Matrigel, or CII-sponges for 14 days with or without chondrogenic induction. * or ^#^ represented *p* ≦ 0.05, and *** or ^###^ represented *p* ≦ 0.001.

To assess the chondrogenic differentiation of TSPCs in CII-sponges *in vitro*, we detected the chondrogenic differentiation-related genes expression of TSPCs after 7- or 14-days culture with or without chondrogenic induction. Cells cultured in monolayer or Matrigel were as control (Figure 3).

Results found that when TSPCs were cultured *in vitro* for 7 days with or without chondrogenic induction, CII-sponges could overall induce TSPCs to differentiate toward chondrogenic lineage. To be more specific, CII-sponges showed upregulated chondrocyte-specific genes expression, including Sox 9 and aggrecan, but downregulated Col1α1 significantly in comparation to Matrigel and monolayer culture. There was no significant difference in col2α1 expressions in three groups with or without induction.

When turned to 14 days culture, we could see that the trends of four genes were overall similar with 7 days culture under completed medium. However, different trends were seen when cells were cultured under chondrogenic medium. The trends of Aggrecan and col2α1 in all of three groups remained similar with 7-day induction. However, the expression of Sox 9 and Col1α1 both in CII-sponges and in Matrigel decreased significantly compared with that in monolayer culture.

However, it is worthy to mention that once TSPCs were cultured under chondrogenic medium either for 7 or 14 days, Col1α1 mRNA levels increased in all of three groups compared with those without induction, indicating that chondrogenic medium could not reverse the fibrochondrogenic differentiation of TSPCs *in vitro*, which is consistent with our previous published data (Qin et al., 2020).

### The Chondrogenic Differentiation of TSPCs *in vivo*

**FIGURE 4:**
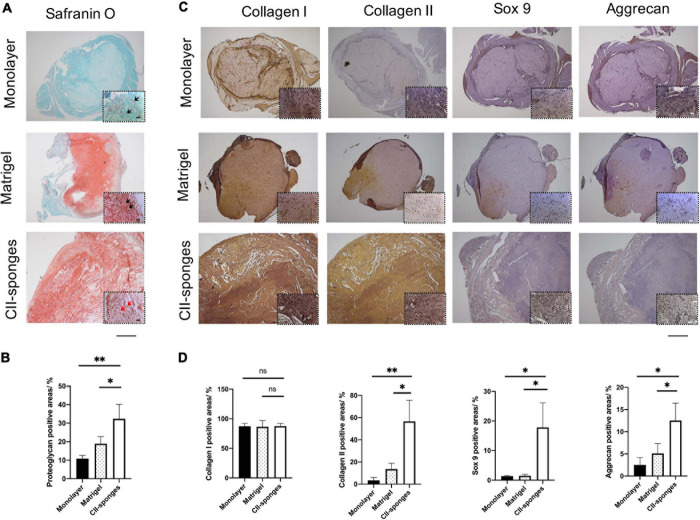
Histological evaluation of the chondrogenic differentiation of TSPCs seeded in Monolayer, Matrigel, or CII-sponges. **(A)** Safranin O/Fast Green staining were applied to observe cells and proteoglycan expression in formed tissues. Scale bar 200 μm and scale bar 20 μm (The magnified photos in dot lines). **(B)** The proteoglycan positive areas were measured by Image ProPlus. ns, represented as no significance; * represented as *P* < 0.05, ** represented as *P* < 0.01, and *** represented as *P* < 0.001. **(C)** Immunohistochemistry staining was also used to confirm the expression of chondrogenic differentiation related proteins, including type I and type II collagen, Sox 9, and aggrecan, in continuous slices. Scale bar 200 μm and scale bar 20 μm (The magnified photos in dot lines). **(D)** The positive areas of these above proteins were quantified by Image ProPlus. ns, represented as no significance; * represented as *p* ≦ 0.05 and ** represented as *p* ≦ 0.01.

To further evaluate the role of CII-sponges in the chondrogenic differentiation of TSPCs *in vivo*, cells were seeded in CII-sponges or Matrigel or in pellet formed by cells in monolayer culture and then induced in chondrogenic medium for 14 days before being transplanted subcutaneously into immunocompromised mice. Safranin O/Fast Green staining was applied to observe cells and proteoglycan expression in formed tissues (Figure 4A). After 5 weeks *in vivo*, a lot of cells could be found in all of three groups, while expression of proteoglycan showed huge differences, and the expression of proteoglycan in CII-sponges groups increased significantly in comparison with that in both monolayer and Matrigel groups (Figure 4B). Cells in both Matrigel and CII-sponges expressed a good deal of proteoglycan (red), but those cultured in monolayer expressed weakly, mostly around cells. It is worth noting that there was a significant difference in cell morphology in CII-sponges compared with the other two groups in magnified photographs. Cells with CII-sponges showed round or oval shape (black arrows), which were more like fibrochondrocytes or chondrocytes, whereas cells in the other two groups still exhibited shutter or long morphology (red arrows), which were more like fibroblasts.

Immunohistochemical staining was also used to confirm the expression of chondrogenic differentiation related proteins, including collagen I and II, Sox 9, and Aggrecan (Figure 4C), and the collagen II, Sox 9 and aggrecan proteins levels in CII-sponges group increased sigificantly in comparison with those in both monolayer and Matrigel groups, while no significant difference in collagen I protein expression in all of three groups (Figure 4D). In all of three groups, strongly positive type I collagen staining could be seen. However, only CII-sponges could induce chondrogenic proteins expression of TSPCs *in vivo*, as evidenced by strong positive type II collagen staining as well as positive Sox 9 and aggrecan staining. Although there was only a small amount of expression of Sox 9 and aggrecan staining around cells in CII-sponges under magnified field by microscopy, none could be found in monolayer or Matrigel groups.

### Histological Evaluation of the Regeneration of Fibrocartilage Transition Zones at BTJ

**FIGURE 5:**
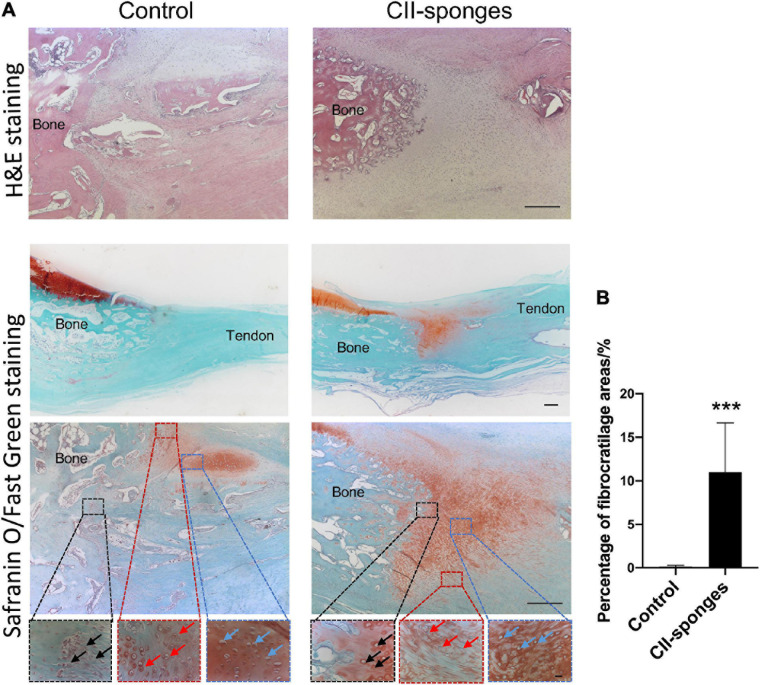
Histological evaluation of the regeneration of fibrocartilage transition zones at BTJ. **(A)** The regeneration of fibrocartilage transition zones at patellar-patellar tendon junction after partial patellectomy was evaluated by histology staining, H&E, Scale bar 200 μm. The whole bone tendon junction, the positive proteoglycan expression areas (red) and the morphology of the cells at the BTJ were observed by Safranin O/Fast Green staining, Scale bar 500 μm (top), Scale bar 200 μm (middle) and Scale bar 20 μm (bottom). In order to observe the morphology of the cells at the fibrocartilage zones, three different regions (Dotted boxes) at BTJ were chosen. To be more specific, in the control group the black box indicated the junction between tendon and bone tissues, fibroblast-like cells could be seen (black arrow), the red box suggested the junction between bone and new formed cartilage, chondrocyte-like cells were found (red arrow), and the blue box represented the new formed cartilage, typical chondrocytes could be observed (blue arrow). In the CII-sponge group, the black box indicated the junction between the bone and new formed cartilage, the red box suggested the junction between the tendon and new formed cartilage, and the blue box represented the new formed cartilage. The chondrocytes could be found in all of these three different areas, and indicated by black, red and blue arrows, respectively. **(B)** Quantification of proteoglycan positive areas in Safranin O staining by Image ProPlus. *** represented as *p* ≦ 0.001.

To further examined if CII-sponges could promote the regeneration of FCs after surgical re-attachment of tendon and bone, histological staining, H&E, and Safranin O, was performed (Figure 5). Based on H&E staining and Safranin O/Fast Green staining, obvious tissue integration was found at BTJ after 8 weeks in both groups. In the control group, more indirect insertion-like structures (also called fibrous entheses) formed at the junction as evidenced by the fact that tendon tissues reconnected with bone directly and most of fibroblast-like cells could be found in the surrounding (black arrows) (Figure 5A). A small new formed cartilage could be observed at BTJ evidenced by positive proteoglycan staining by Safranin O/Fast Green staining and round or oval chondrocyte-like cells (red arrows and blue arrows) could be seen in this new-formed cartilage (Figure 5A). However, in CII-sponges group, fibrocartilaginous entheses formed, characterised by four gradual transition zones, including bone, a large amount of cartilage-like tissues, and tendon, where typical chondrocyte-like cells (black, red, and blue arrows) could be found in these three areas (Figure 5A). Cartilage-like tissues expressions were qualified by measuring the areas of positive proteoglycan in Safranin O/Fast green staining using Image ProPlus, which showed the positive area of proteoglycan in CII-sponges group was significantly larger than that in control group, ^∗∗∗^*p* ≦ 0.001 (Figure 5B).

## Discussion

New fibrocartilage zone formation is one of the most important indicators of good BTJ reconstruction (Wong et al., 2004; Zhang M. et al., 2019; Zhang Y. et al., 2019; Shi et al., 2020). It is well known that the regeneration of fibrocartilage is a slow and difficult process after tendon reattached to bone. It is reported that the mechanical strength of BTJ repair without FCs formed was only one-tenth of that of native patellar-patellar tendon junction (Lui P. et al., 2010). Although it is believed that cartilage-like tissue formation or the presence of chondrocytes at the BTJ is an intermediate process for the regeneration of FCs (Lui P.P. et al., 2010), it cannot be denied that this process is the most important one for better BTJ healing. Lu et al. (2008) confirmed that the cartilage to tendon was superior to bone to tendon healing, which might be due to the fusion of cartilage to tendon is much easier than that of bone to tendon. It is reasonable to believe that the key to promote the regeneration of FCs is to promote the chondrogenesis of MSCs migrated from bone or tendon and hence to promote the new formation of cartilage-like tissue at the early healing stage. Indeed, Wong et al. (2004, 2009) have already demonstrated that interposition of autologous articular cartilage or allogenic chondrocyte pellet could improve FC regeneration using the partial patellectomy rabbit model. All of these findings suggest that promoting the chondrogenesis of stem cells and hence new cartilage formation at BTJ is not only a key process but also one of the effective means for better reconstruction of BTJ.

We previously found that TSPCs differentiated to fibrochondrocytes under chondrogenic induction, in this study we further examined if CII-sponges we made would guide TSPCs to adopt chondrogenic phenotypes and eventually promote the formation of fibrocartilage tissues. Data showed that CII-sponges with good porosity and good cell compatible were beneficial for chondrogenesis of TSPCs *in vitro* or *in vivo*. Moreover, interposition of CII-sponges at the BTJ improved the fibrocartilage formation in the rabbit PP model.

Pellet culture system is the most classical culture system used to assess the chondrogenic differentiation of stem cells. Although most of studies on tendon stem cells have confirmed that tendon stem cells had the chondrogenic potential in pellet culture system, but these reports generally only detect the expression of proteoglycan by safranin O staining or alcian blue staining (Bi et al., 2007; Rui et al., 2010; Zhang and Wang, 2010). There was only a little amount of expression around cells in most studies, and no further observation into cartilage differentiation indicators, such as collagen I/II, aggrecan and other chondrocyte-related proteins was conducted. In our previous study, we found that tendon stem cells in a monolayer culture differentiated to fibrocartilage cells rather than chondrocytes by detecting expression of collagen I and II, Sox 9, and tenascin C. Similar results were found in this study when TSPCs were cultured in the 3-D environment. Even though the mRNA level of Col1α1 was downregulated when TSPCs were seeded in Matrigel or CII-sponges, strong expression of collagen I could be found, as evidenced by IHC staining.

Collagen scaffolds are usually manufactured for surgical and dental purposes or cell culture matrices. The porous collagen sponges provide a highly biocompatible environment for cells due to their high porosity and interconnected pores. Porosity and pore sizes have been demonstrated to regulate cellular behaviours (Loh and Choong, 2013; Zhang et al., 2014), like adhesion and ingrowth. This high biocompatibility makes collagen sponges perfect implantable medical products and scaffolds for *in vitro* testing systems. In our study, the porosity of CII-sponges we made was over 99% porous, and interconnected, round, or elongated pores could be seen (Qin et al., 2010). Good porosity and the interconnected pores are important in diffusing nutrients and gases as well as removing metabolic waste. Pore size of scaffolds can also influence cellular attachment, morphology, and differentiation (Matsiko et al., 2015). For chondrocytes, pores of 50–300 μm are usually recognised as the suitable sizes for stimulating stem cell chondrogenesis and cartilage regeneration (Nehrer et al., 1997; Zhang et al., 2014). Pore size of CII-sponges we made was approximately 92.17 ± 29.55 μm (Qin et al., 2010). CII-sponges could also facilitate the adhesion of TSPCs, evidenced by a large proportion of live cells and a few dead cells found after 14-day culture. AlamarBlue confirmed that cells adhered to CII-sponges also remained a stable viability over time. PKH67 *in vitro* tracking data showed that under chondrogenic induction, more cells expressed green fluorescence compared with those without induction.

Many studies believed that the differentiation lineage of stem cells could be directed by the composition of the extracellular matrix (Badylak, 2005; Evans et al., 2010). For example, Bosnakovski et al. (2006) have found that type II collagen hydrogels significantly induced chondrogenic differentiation of bone marrow mesenchymal stem cells compared to type I collagen hydrogels or alginate gels. Buma et al. (2003) compared the role of type I and type II collagen matrices for the repair of full-thickness articular cartilage defects and found that type I collagen were superior to guide progenitor cells from a subchondral origin into the defect areas whereas type II collagen matrices were better in directing stem cells into a chondrocyte phenotype. In our study, we found that CII-sponges have the capability of promoting the differentiation of TSPCs down the chondrogenic lineage as evidenced by gene expression *in vitro*. Chondrocyte-related genes, including Sox 9, the early marker of chondrogenesis, as well as col2α1 and aggrecan, the late markers, were all upregulated by CII-sponges with or without chondrogenic induction at both time points. However, Matrigel did not show similar effects, with only col2α1 and aggrecan upregulated after 14-day chondrogenic induction. Meanwhile, compared with monolayer groups, the expression of fibroblastic marker, col1a1, decreased overall in both Matrigel groups and CII-sponges groups, with only slightly increased in Matrigel groups after 7-day chondrogenic induction. This might confirm the hypothesis that stem cell differentiation could be driven by the matrices specific to a particular tissue.

Type II collagen predominantly composed of articular cartilage, thus a scaffold made with type II collagen might be suitable for cartilage-tissues formation by directing MSCs differentiation toward chondrocytes. Chen et al. (2011) observed the role of type II collagen sponge materials on promoting cartilage repair using MRI combined with histology. Funayama et al. (2008) indicated that type II collagen gel was suitable for injection into cartilage defects and offered a useful scaffold during chondrocyte transplantation.

Immunohistology observations from *in vivo* data revealed that CII-sponges promoted the proteoglycans deposit as well as the expression of chondrogenic specific markers, including Sox 9, aggrecan, and type II collagen, whereas Matrigel only induced a little of these three markers expression, despite of a large amount of proteoglycans deposit similar as CII-sponges.

Noticeably, compared with non-induction groups, the mRNA and protein levels of fibroblastic marker, type I collagen, increased in all of three groups after chondrogenic induction. This is consistent with our previous results which found TSPCs differentiate to fibrochondrocytes rather than chondrocytes. Type I collagen is one of the most important protein to distinguish these two cells. Although CII-sponges and Matrigel downregulated the mRNA level of col1a1 with or without induction, IHC staining showed that type I collagen protein deposits could be found in both scaffolds. This might indicate that TSPCs differentiate toward fibrocartilage cells under chondrogenic induction when they were cultured in two-dimensional monolayer culture system or in three-dimensional scaffolds.

Cartilage-like tissues could be found during healing at BTJ injured model in CII-sponges group. Although, it is reported that endochondral ossification occur subsequently and these cartilaginous tissues disappear eventually during healing (Lui P.P. et al., 2010). The presence of chondrocytes at the BTJ was commonly associated with better healing. In our study, CII-sponges facilitated the formation of cartilage-like tissues with obvious four transition zones and abundant of proteoglycan expression. This continuous change in tissue composition from tendon to bone is presumed to aid in the efficient transfer of load between the two materials. However, no obvious fibres inserted to bone were found in CII-sponges group. The reason for this may be the time point we chose, and collagen fibres occurred in a relatively late time point, like at 18 or 24 weeks, and then sharpey’s fibres formed.

In conclusion, we found that CII-sponges we made facilitated TSPCs to adopt more chondrocyte phenotype *in vitro* and *in vivo* and meanwhile promoted the regeneration of fibrocartilage-like tissues in the BTJ injured model. This CII-sponges might well be suitable for the reconstruction of FC at BTJ.

## Data Availability Statement

The original contributions presented in the study are included in the article/supplementary material, further inquiries can be directed to the corresponding author/s.

## Ethics Statement

The animal study was reviewed and approved by Guangzhou Red Cross Hospital Ethics Committee.

## Author Contributions

SQ and JX drafted the manuscript. SQ, WW, PH, WM, LC, JZ, XX, ZL, PW, QM, and FD performed the experiments. SQ analysed the data. AL, HC, and XH contributed the experiment instruments and analysis tools. SQ and JX conceived and designed the experiments. All authors contributed to the article and approved the submitted version.

## Conflict of Interest

The authors declare that the research was conducted in the absence of any commercial or financial relationships that could be construed as a potential conflict of interest.
